# Update on the mechanism of microglia involvement in post-stroke cognitive impairment

**DOI:** 10.3389/fnagi.2024.1366710

**Published:** 2024-06-03

**Authors:** Tianxiang Zeng, Jun Liu, Wenjun Zhang, Yanyan Yu, Xinyun Ye, Qianliang Huang, Peng Li, Qiuhua Jiang

**Affiliations:** ^1^Department of Neurosurgery, The Affiliated Ganzhou Hospital, Jiangxi Medical College, Nanchang University, Ganzhou, Jiangxi, China; ^2^Department of Neurosurgery, The 2^nd^ Affiliated Hospital, Jiangxi Medical College, Nanchang University, Nanchang, Jiangxi, China; ^3^Department of Recovery Medicine, The Affiliated Ganzhou Hospital, Jiangxi Medical College, Nanchang University, Ganzhou, Jiangxi, China; ^4^Department of Neurosurgery, Institute of Brain Diseases, Nanfang Hospital of Southern Medical University, Guangzhou, China

**Keywords:** PSCI, microglia, neuroinflammation, stroke, signaling pathway

## Abstract

Post-stroke cognitive impairment (PSCI) is a clinical syndrome characterized by cognitive deficits that manifest following a stroke and persist for up to 6 months post-event. This condition is grave, severely compromising patient quality of life and longevity, while also imposing substantial economic burdens on societies worldwide. Despite significant advancements in identifying risk factors for PSCI, research into its underlying mechanisms and therapeutic interventions remains inadequate. Microglia, the brain’s primary immune effector cells, are pivotal in maintaining, nurturing, defending, and repairing neuronal function, a process intrinsically linked to PSCI’s progression. Thus, investigating microglial activation and mechanisms in PSCI is crucial. This paper aims to foster new preventive and therapeutic approaches for PSCI by elucidating the roles, mechanisms, and characteristics of microglia in the condition.

## 1 Introduction

Post-stroke cognitive impairment (PSCI) is a clinical syndrome characterized by cognitive deficits. PSCI can be categorized into two types based on the severity of cognitive deficits: post-stroke cognitive impairment without dementia and post-stroke dementia (Wang and Dong, 2021). Vascular cognitive impairment (VCI) is a broader concept, initially introduced by Professor Hachinski, that encompasses all cognitive deficits potentially caused by vascular factors (Skrobot et al., 2017). It highlights the critical role of vascular factors in cognitive decline. PSCI is a significant subtype of VCI, with the primary distinction being that PSCI specifically denotes cognitive deficits triggered by stroke events, whereas VCI encompasses cognitive deficits associated with clinical or imaging evidence of cerebrovascular disease (Wang and Dong, 2021). A systematic review and meta-analysis revealed that over a third of stroke survivors experience cognitive deficits, with the prevalence exceeding 50% in some countries (Barbay et al., 2018). Furthermore, it has been increasingly observed that the cognitive function of individuals with PSCI deteriorates over time, becoming more pronounced with age (Huang et al., 2023). PSCI severely impacts patients’ physical and mental well-being and increases the social and economic burden. The underlying mechanisms of PSCI remain largely unclear, and effective treatments to halt its progression are still lacking. Research has historically focused on risk factors such as cerebral ischemic injury, neurodegeneration, neuroinflammation, blood–brain barrier (BBB) disruption, and oxidative stress (Sayed et al., 2020; Wang et al., 2022; Tack et al., 2023; Zhang et al., 2023; Jiang et al., 2024). Recently, the role of microglia in PSCI has garnered increasing attention (Tack et al., 2023). Microglia, the brain’s primary immune and pro-inflammatory cells, are critical to the central nervous system’s (CNS) immune response. Evidence suggests that microglia-mediated neuronal damage and dysfunction play a pivotal role in the pathogenesis and progression of PSCI, involving neuropathological changes post-stroke and several signaling pathways implicated in cognitive deficits, such as TLR4, p25/CDK5, Nuclear factor kappa-B (NF-κB), and CX3CR1 (Wang et al., 2022; Yu et al., 2023; Ge et al., 2024). Understanding the role and activation mechanisms of microglia in PSCI and exploring intervention strategies targeting these mechanisms are crucial. This article reviews the activation mechanisms of microglia and their impact on the prognosis of PSCI, offering new insights into the prevention and treatment of PSCI.

## 2 Features of microglia

Microglia were first identified by the Spanish neuroscientist Pío del Río Hortega in 1919. Over the ensuing century, extensive research has been conducted on microglia and their association with central nervous system (CNS) diseases (Prinz et al., 2019). Initially, due to their similar characteristics and immune phenotypes to monocytes and macrophages, microglia were thought to originate from the hematopoietic system (Eglitis and Mezey, 1997). However, subsequent research, notably by Ginhoux et al. (2010) established that microglia are derived from primitive macrophages in the embryonic yolk sac during development. Under normal physiological conditions, microglial formation and nerve growth are integral to the brain development process. Microglia exhibit versatility under various stimulations, manifesting in diverse forms that are critical for understanding embryonic origin, central system development, and pathological changes (Nayak et al., 2014). Typically, microglia display a ramified morphology, crucial for maintaining CNS homeostasis (Masuda et al., 2011). In pathological states, disruptions to brain homeostasis trigger microglial activation, leading to the production of cytokines and phenotypic transformations. These changes result in different polarized phenotypes, each responding to specific types of pathological alterations (Gülke et al., 2018; Rajan et al., 2019). The existing classic theory divides activated microglia into two main phenotypes: M1 and M2. M1 activation induces inflammation and neurotoxicity, while M2 activation elicits anti-inflammatory and repair responses (Prinz et al., 2019). Therefore, microglia serve as a “double-edged sword” maintaining brain homeostasis through opposing pathophysiological effects. However, recent studies suggest that microglial phenotypes may be more diverse than previously thought (Dubbelaar et al., 2018). In fact, pro-inflammatory and anti-inflammatory cytokines do not represent absolute opposites. Microglia may exhibit a range of intermediate phenotypes between M1 and M2, challenging the current simplistic dichotomous classification. Microglia adapt to complex environments through their diverse phenotypes, ultimately exerting a protective role. Given their involvement in synaptic development, maintenance, apoptosis, and other processes crucial for brain protection and homeostasis, microglial dysfunction is increasingly linked with neurological diseases (Nayak et al., 2014; Yang et al., 2020). Previous studies have demonstrated that ischemic stroke triggers a cascade of microglial activation events, including morphological changes, proliferation, and polarization (Zhang, 2019). Notably, during the activation process, morphological alterations in microglia become pronounced as blood flow decreases. Initially, they exhibit debranching-like changes, followed by either shrinking or extending branches, and ultimately transforming into a macrophage-like amoeba shape (Davalos et al., 2005). The extent of these morphological changes not only reflects functional changes in microglia but also correlates with the size and distribution of ischemic lesions within the brain. In the middle cerebral artery occlusion model of cerebral ischemia, Li et al. (2013) observed that activated microglia were concentrated in the ischemic area of the brain, which can be roughly divided into three areas: core, accumulation, and marginal areas. The morphology and number of microglia vary across different regions, which may be attributable to the origin and sensitivity of microglia in these regions. Previous studies have found that after ischemic stroke, microglia can originate from three different sources: local proliferation, exogenous cell infiltration, and progenitor cell mobilization (Morrison and Filosa, 2013). Microglia from different sources exhibit different functional properties, and the activation of microglia in the striatum is more complex and variable than in the cortex (Dubbelaar et al., 2018). Furthermore, compared with severe ischemia, transient ischemia causes only a mild reduction in microglia numbers, and this reduction can be reversed after reperfusion (Li et al., 2013). These observations highlight the unique spatial and temporal characteristics of microglia (Perego et al., 2011; Li et al., 2013; Morrison and Filosa, 2013).

## 3 How stroke activates microglia

### 3.1 Activation of microglia through inflammatory response

**Figure 1:**
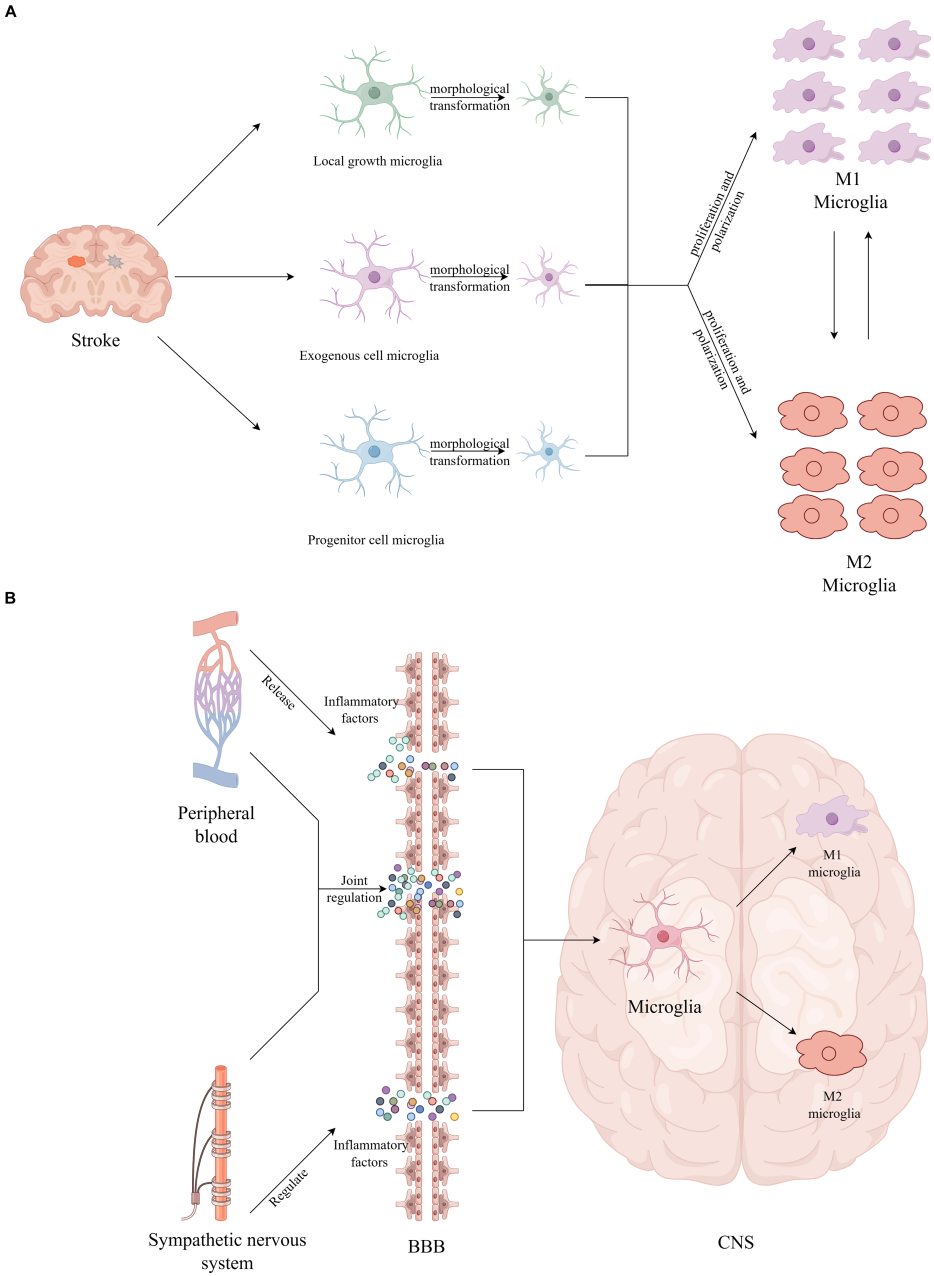
**(A)** Stroke provokes microglial activation, including morphological transformation, proliferation, and polarization. **(B)** The inflammatory factors produced by peripheral inflammation cross the BBB and directly activate microglia.

The BBB is a crucial protective barrier within the brain, offering protection against potentially harmful foreign substances under physiological conditions. Similarly, microglia, as pivotal immune cells in the CNS, play an essential role in various physiological and pathological processes, including CNS development, maintenance of homeostasis, and cognitive impairment (Prinz et al., 2019). After stroke, multiple mechanisms such as oxidative stress, neuroinflammation, and immune responses compromise the integrity of the BBB’s tight junctions. This compromise leads to a diminished capacity to block harmful substances, ultimately accelerating the progression of neurological dysfunction (Yang et al., 2019; Li et al., 2024). Quan (2014) and Galic et al. (2012) discovered that the activity of the sympathetic nervous system regulates CNS functions by fostering inflammation. This regulation may be attributed to the influence of cytokine transport on vascular structure and function, which in turn, triggers secondary organ complications (Xu et al., 2016). Stroke patients often face multiple complications, such as pneumonia and stress ulcers, which lead to immune dysregulation and trigger systemic immune suppression. Inflammatory factors produced by peripheral inflammation can reach the CNS through peripheral circulation and cross the compromised BBB, thereby activating microglia (Belevych et al., 2010; Hoogland et al., 2015; Liu et al., 2016; Teng et al., 2017). Activated microglia exhibit dynamic changes as the stroke progresses (Hu et al., 2012). Moreover, damaged brain tissue post-stroke secretes various inflammatory mediators, including cytokines, chemokines, and reactive oxygen species (ROS) (Xiong et al., 2016). During activation, microglia can polarize towards either a pro-inflammatory (M1) phenotype or a protective (M2) phenotype. M1 microglia contribute to neuronal damage by releasing inflammatory mediators like TNF-α, IL-6, IL-1β, and IFN-γ, creating a positive feedback loop that exacerbates inflammation. This cascade intensifies harmful circulation, compromises the BBB’s integrity, induces brain edema, and accelerates the influx of inflammatory cells, leading to neuronal damage (Dong et al., 2021; Li et al., 2022). Conversely, M2 microglia secrete anti-inflammatory factors, such as IL-4, IL-10, and IL-13, which inhibit inflammation, clear cellular debris, and promote angiogenesis, stabilizing the disease process (Alshammari et al., 2023). In exploring the pro-inflammatory effects of M1 microglia, Alshammari et al. (2023) and Qin et al. (2022) also highlighted the significant potential of M2 microglia in promoting brain tissue repair and functional regeneration through the release of neurotrophic factors. Ricci and Liesz (2023) discovered that the immune system could mitigate the inflammatory response in the early stages of a stroke and modulate microglial function by regulating other immune cell types. The intertwined effects of central system inflammation and peripheral inflammation alter neuronal excitability, leading to the onset and exacerbation of PSCI (Hoogland et al., 2015). Hence, moderating the excessive inflammatory response of microglia is crucial for effective stroke treatment and recovery (see Figure 1).

### 3.2 Activation of microglia by ROS

**Figure 2:**
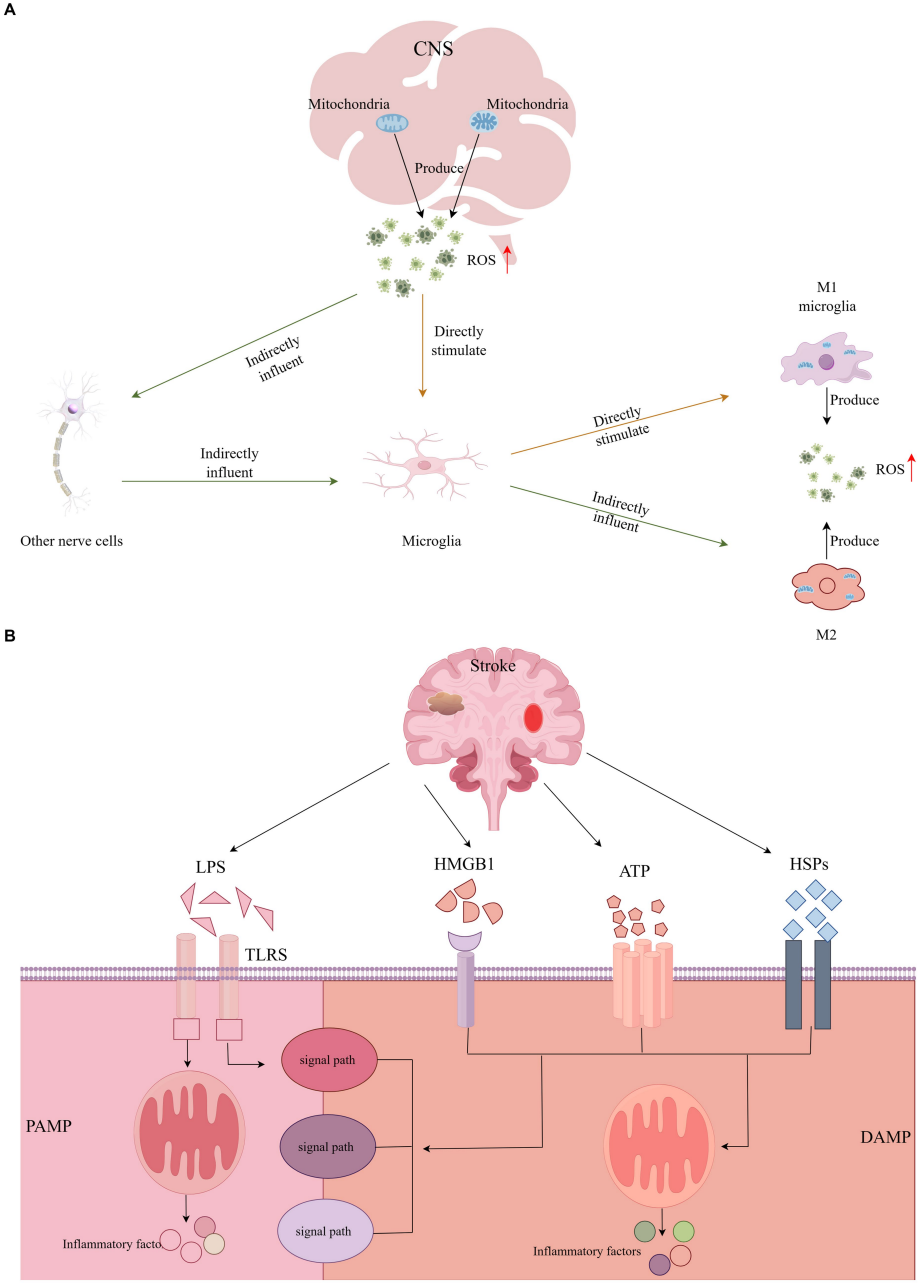
**(A)** The central nervous system directly or indirectly activates microglia through the production of ROS, and activated microglia continue to produce large amounts of ROS. **(B)** DAMP or PAMP activates a variety of different signaling pathways to activate microglia and induce inflammatory responses.

In humans, mitochondria are the principal generators of ROS, which can activate microglia directly or indirectly through the impact on other CNS cells (Block et al., 2007). Previous research has demonstrated that the upregulation of microglial activation can be mitigated by antioxidants, indicating that suppressing ROS significantly diminishes cell proliferation and microglial activation (Roy et al., 2008; Zou et al., 2010). Throughout the cerebral ischemia–reperfusion cycle, activated microglia persistently produce substantial amounts of ROS (Pawate et al., 2004). In such an environment rich in ROS, microglia are inclined to polarize towards the M1 phenotype, diminishing M2 activation and thereby intensifying the inflammatory response (Zhu et al., 2022). Moreover, ROS serves as a critical signaling molecule, contributing to mitochondrial dysfunction and neuronal damage (Nair et al., 2011). This involvement in pathophysiological processes underpins cognitive impairment. The exacerbation of cognitive dysfunction, progressing towards PSCI, is further fueled by microglial mitochondrial dysfunction and the overactivation of sources of ROS in the CNS, such as reduced nicotinamide adenine dinucleotide phosphate oxidase and xanthine oxidase. This results in a disturbance in intracranial homeostasis, leading to a cascade of inflammatory and oxidative reactions (Block et al., 2007; Ronaldson and Davis, 2020). Additionally, ROS-induced endothelial damage significantly compromises the integrity of the BBB, weakening its protective role and facilitating the entry of detrimental substances, which is another critical factor contributing to cognitive deficits (Colonna and Butovsky, 2017). Given the pivotal role of ROS in microglial activation, neuroinflammation, and BBB integrity, targeting ROS dynamics may emerge as a novel therapeutic strategy (see Figure 2).

### 3.3 Activation of microglia by pathogen associated molecular pattern (PAMP) or damage associated molecular pattern (DAMP)

As stroke progresses, neuronal damage or necrosis triggers the release of endogenous danger signals by the affected cells within the CNS, known as Damage-Associated Molecular Patterns (DAMPs). Concurrently, peripheral inflammation and disruptions in the BBB allow for the invasion of pathogens, which release Pathogen-Associated Molecular Patterns (PAMPs) (Ronaldson and Davis, 2020). Microglia, acting as sentinels of CNS homeostasis, respond to these signals by recognizing either DAMPs or PAMPs (Colonna and Butovsky, 2017). This recognition initiates various signaling pathways that lead to microglial activation, culminating in an inflammatory response and cell death (Jackson et al., 2020). Notably, the High Mobility Group Box 1 protein (HMGB1) is identified as a quintessential DAMP, playing a pivotal role in inflammasome activation and the regulation of apoptosis. Furthermore, Heat Shock Proteins (HSPs), Lipopolysaccharides (LPS), and Adenosine Triphosphate (ATP) are also significant in these processes, contributing to the cascade of inflammatory and apoptotic events following a stroke (Teng et al., 2017).

## 4 The mechanism of microglia involvement in PSCI

### 4.1 Toll-like receptor (TLRs) signaling pathway in PSCI

Neuroinflammation typically refers to a specific immune response triggered by microglia and astrocytes within the CNS, prevalent across a spectrum of pathological conditions such as infections, trauma, and ischemia (Gao et al., 2023). This response bifurcates into acute and chronic phases of neuroinflammation. The acute phase is commonly observed during the initial stages of trauma repair, while the chronic phase is frequently associated with a range of neurodegenerative disorders, including PSCI, Alzheimer’s disease, Parkinson’s disease, among others (Lecca et al., 2022). A pivotal study demonstrated that attenuating symptoms of PSCI by silencing microglia to suppress neuroinflammation underscores the critical role of microglia-induced neuroinflammation in PSCI (Jackson et al., 2020). The role of microglia in releasing inflammatory mediators via the Toll-like receptor (TLR) signaling pathway is a key area of investigation. Toll, a pattern recognition receptor (PRR), initially identified during the study of *Drosophila melanogaster* embryonic development, engages in signal transduction upon binding to its ligands. This interaction regulates immune-related inflammatory responses and is instrumental in neural processes, contributing significantly to the pathogenesis and progression of degenerative diseases (Lehnardt, 2010). TLR4, a prominent PRR on microglia surfaces, regulates their immune and inflammatory responses. Research indicates TLR4’s involvement in recognizing Damage-Associated Molecular Patterns (DAMPs) post-stroke. During the inflammatory response, the extracellular release of HMGB1 as an endogenous TLR4 ligand activates and amplifies the inflammatory response *in vivo*, intensifying secondary brain damage and precipitating PSCI development in mice (Sayed et al., 2020; Wang et al., 2021). Experimental studies on stroke-induced chronic hypoperfusion revealed a marked increase in HMGB1 expression levels within the cerebral cortex and hippocampus. Treatments targeting TLR4, such as the receptor inhibitor TAK-242 or intravenous administration of a neutralizing HMGB1 antibody, not only reduced microglial activation and inflammatory cytokine expression but also decreased neuronal death in the hippocampus, thereby mitigating cognitive impairment in mice (Matsunaga et al., 2011; Vidyanti et al., 2020). Furthermore, elevated TLR4 expression in the brain tissue and peripheral blood of Alzheimer’s disease patients has been linked to the progression of cognitive impairments (Miron et al., 2018).

### 4.2 Janus kinase-signal transducer and activator of transcription (JAK–STAT) pathway in PSCI

The Janus Kinase 2-Signal Transducer and Activator of Transcription 3 (JAK2-STAT3) signaling pathway is central to the regulation of inflammation, apoptosis, immune responses, and various cellular functions. Research highlights its crucial role in the activation and phenotypic transition of microglia, particularly in the context of stroke. Typically, activated microglia transition from the pro-inflammatory M1 phenotype to the anti-inflammatory M2 phenotype, a change that mitigates chronic ischemic damage to white matter, fosters white matter reconstruction, and enhances cognitive performance (Chen et al., 2017; Wang et al., 2021). The JAK kinase is part of the non-receptor tyrosine kinase family, with STAT serving as both a substrate and a downstream signaling component of JAK. Following cerebral injury, pro-inflammatory cytokines stimulate the phosphorylation of STAT3 by JAK kinase. The phosphorylation of the Y705 tyrosine residue on STAT3 is implicated in the microglia-mediated inflammatory process (Meng et al., 2016). Studies on stroke have shown increased phosphorylation levels of JAK2 and STAT3 in the cortical and striatal regions of rat brains. The use of AG490, an inhibitor of JAK2 tyrosine kinase, has been shown to decrease infarct size, lower the number of apoptotic cells, and improve neurological outcomes (Satriotomo et al., 2006). Moreover, under normal physiological conditions, microglia remain in a non-activated state, largely due to neuronal regulation through contact-dependent mechanisms. Stroke disrupts this regulatory interaction, leading to abnormal microglial activation. Post-stroke, neurons release soluble Fas ligand (sFasL), which activates the phosphorylation cascade of the JAK2/STAT3/NF-κB pathway. This activation promotes M1 microglial polarization, igniting inflammation, causing neuronal damage, and consequently contributing to cognitive decline (Niu et al., 2012; Meng et al., 2016).

### 4.3 NF-κB pathway in PSCI

**Figure 3:**
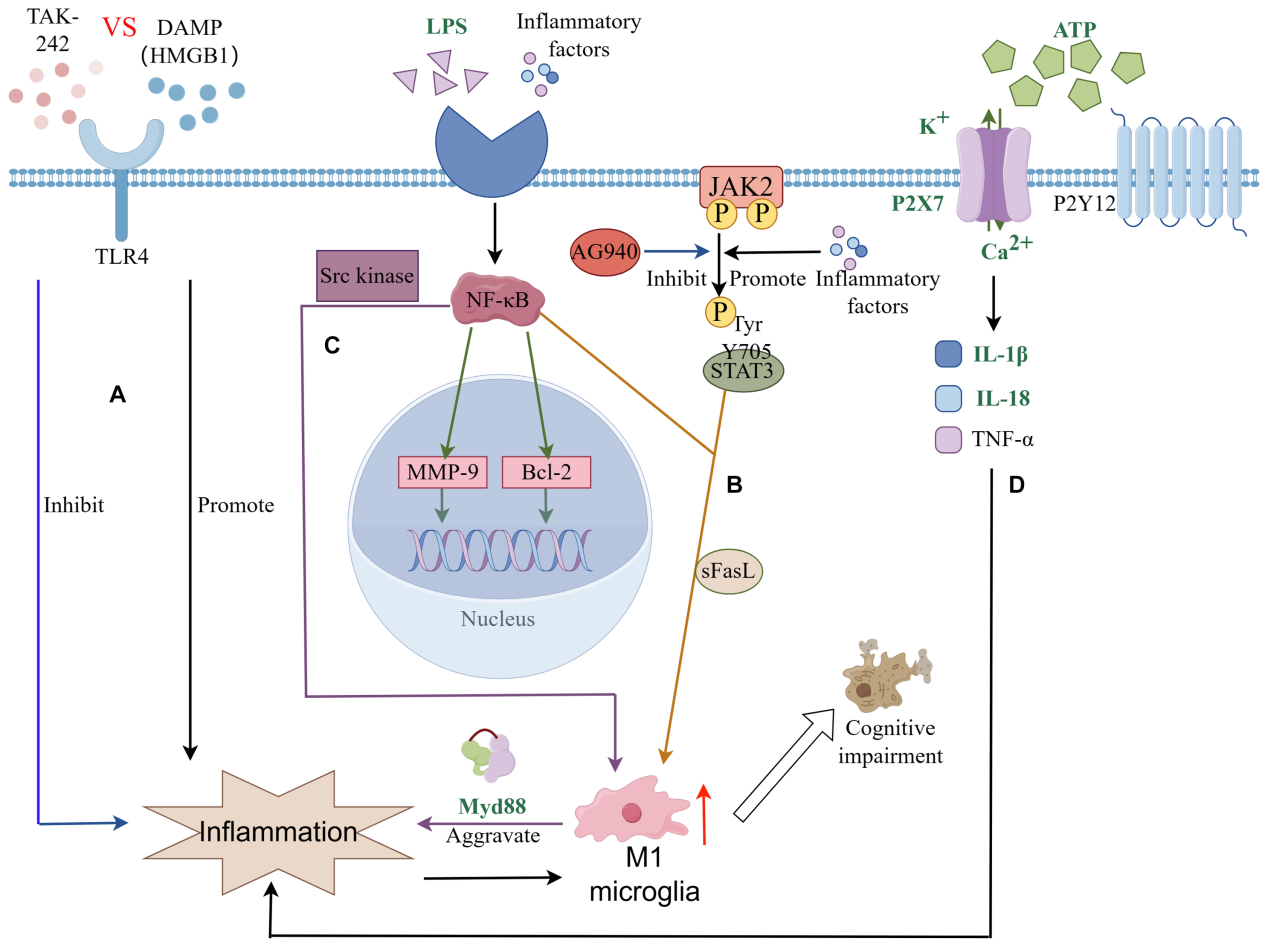
Microglia connect signaling pathways in PSCI through neuroinflammation. **(A)** TLRs signaling pathway is involved in microglia-mediated neuroinflammation; **(B)** JAK–STAT signaling pathway plays an important role in microglial polarization and neuroinflammation; **(C)** NF-κB signaling pathway plays an important role in microglial polarization and neuroinflammation. Plays a key role in microglial inflammation and neuronal apoptosis; **(D)** P2 receptor family signaling and the inflammatory response of microglia.

NF-κB is a key transcription factor within the classical signaling pathway, primarily consisting of the kappa B (IκB) protein and the NF-κB dimer. Clinical studies and animal research have established the critical role of the classical NF-κB signaling pathway in ischemic stroke-related injuries. Activation of the NF-κB pathway in microglia by stimuli such as TNF-α, LPS, and IL-1β leads to the activation of IκB kinase, which in turn promotes the phosphorylation and subsequent degradation of the IκB protein. This degradation process liberates the NF-κB dimer, facilitating the regulation of gene transcription, including the anti-apoptotic gene bcl-2 and the matrix metalloproteinase (MMP) involved in extracellular matrix remodeling (Liu et al., 2021; Wang et al., 2022; Huang et al., 2023). Further research has revealed that NF-κB activation is a feature of models of PSCI, and that inhibitors of NF-κB can notably enhance cognitive function by moderating cell apoptosis. This modulation of the apoptotic pathway serves to inhibit neuronal apoptosis, thereby ameliorating cognitive deficits post-stroke (Liu et al., 2021; Li et al., 2023). Moreover, NF-κB plays a pivotal role in the neuroinflammatory response mediated by microglia. Specifically, it activates the Src kinase-NFκB pathway in response to High Mobility Group Box 1, prompting a shift in microglia towards M1-type activation. This M1 polarization fosters inflammation and stimulates the release of a plethora of cytokines through the Myeloid Differentiation primary response 88 (MyD88)-dependent pathway, thus intensifying neuroinflammation (Socodato et al., 2015; Mut-Arbona and Sperlágh, 2023) (see Figure 3).

### 4.4 Purinergic P2 receptor family signaling pathway in PSCI

Members of the purinergic receptor family play very important roles in physiological or pathological conditions of the brain. The P2 receptor family mainly includes two types: P2X and P2Y. The P2X receptor is a type of ligand-gated ion channel, while the P2Y receptor is a G protein-coupled receptor. Different types of P2 receptors also include a variety of subtypes, including seven distinct P2X receptors (P2X1, P2X2, P2X3, P2X4, P2X5, P2X6, P2X7) and eight different P2Y receptors (P2Y1, P2Y2, P2Y4, P2Y6, P2Y11, P2Y12,P2Y13 and P2Y14) (Volonté et al., 2006). Many previous studies have found that multiple subtypes in the P2 family are involved in the functional expression of nerve cells and immune cells in CNS diseases, among which P2X7 and P2Y12 have received a lot of attention (Hirayama et al., 2021; Morrone et al., 2021; Illes et al., 2023; Paul et al., 2023). The now better known P2 ligand, ATP, is often released in large quantities in damaged or activated microglia as a signaling molecule. After stroke, due to the imbalance of central system homeostasis, ATP in the internal environment increases significantly along with ischemic and hypoxic events in neuronal cells. The P2X7 receptor is activated by high concentrations of ATP, which in turn induces the production and release of many inflammatory factors. Such as IL-1β, IL-18, and TNF-ɑ (Chu et al., 2012; Rajan et al., 2019; Bahader et al., 2021). Chu et al. (2012) found that after using P2X7 receptor antagonists in a mouse model of cerebral ischemia perfusion, nerve cells, microglia, and inflammatory cytokines were significantly reduced in the hippocampus of mice with cerebral ischemia. At the same time, the cognitive abilities related to learning and memory of mice were improved. Additionally, both Eyo and Cavaliere demonstrated that in regards to the oxygen and glucose deprivation (OGD) model, P2X7 antagonist treatment can regulate microglial death by reducing OGD. These studies suggest that P2X7 receptor inhibitors may be therapeutic Potential targets of PSCI (Cavaliere et al., 2005; Lou et al., 2016). Except P2X7, another important signaling receptor P2Y12 also involved in neurodegenerative diseases (Chu et al., 2012). In the past few decades, antiplatelet drugs such as P2Y12 inhibitors clopidogrel and ticagrelor have played a huge role in the field of cardiovascular stroke. Some scholars believe that they may cause side effects related to cognitive function because they involve the P2Y12 receptor (Xu et al., 2016; Wang et al., 2023). In fact, there is evidence that the P2Y12 receptor may enter the brain and play a potential role due to the destruction of the BBB (Li et al., 2020; Wang et al., 2023). A recent study corroborates that post-stroke clopidogrel intake in mice impairs learning and memory capabilities and lowers survival rates, with BBB integrity compromise cited as a significant factor(Paul et al., 2023).

## 5 Microglia-based approach to PSCI treatment

Based on the above analysis, it is shown that microglia play a key role in the development of PSCI. They are not only a key regulator of brain homeostasis, but also regulated by specific signaling pathways and cytokines. Therefore, exploring treatments that effectively inhibit pro-inflammatory microglia and enhance the anti-inflammatory function of microglia is of great significance to improving the treatment and recovery of PSCI. A recent systematic review and meta-analysis emphasized the importance of the timing of anti-inflammatory interventions, with better PSCI outcomes being achieved by initiating treatment within the first 24 h after stroke induction (Tack et al., 2023). Current clinical research on how to modulate microglia focuses on two main areas: regulating microglial activation and polarization and promoting microglial neuroprotection. The main three drugs with more in-depth clinical trials available include minocycline, fingolimod, and erythropoietin, all three of which emphasize the therapeutic importance of microglia of the M2 phenotype (Bond and Rex, 2014; Hait et al., 2014; Lu et al., 2021). Of course, some drugs have received attention in the past, such as TLR receptor inhibitor TAK242, melatonin, phosphodiesterase type 5 inhibitors etc., (Matsunaga et al., 2011; Moretti et al., 2016; Liu et al., 2019). In a recent study, excessive activation of filaggrin was observed in brain tissues of human and rat cerebral hemorrhage leading to microglial activation releasing neuroinflammation, which affects the progression of PSCI, and the study of how to regulate the filaggrin signaling pathway is also a new therapeutic avenue (Almarghalani et al., 2023). Alshammari et al. (2023) also found that C21, as a selective nonpeptide angiotensin II type 2 receptor agonist, could effectively modulate microglia and macrophage polarization by stimulating the Angiotensin II type 2 receptor, reducing it toward a pro-inflammatory phenotype and ultimately leading to a favorable outcome of PSCI. The role that C21 plays in the PSCI modulation of neuroinflammatory responses in PSCI may indicate the importance of the brain renin-angiotensin system and may be a potential therapeutic target for PSCI in the future. Besides by inhibiting neuroinflammation, neuroprotective agents might also be important in the treatment of PSCI. Zhang et al. (2023) found that DL-3-n-butylphthalid, as a neuroprotective agent, acted as a neuroprotective agent by inhibiting the activation of microglia in ischemia/reperfusion rats to protect and improve neurons, which in turn alleviated PSCI. Of course, in addition to drug therapy, noninvasive treatment modalities such as transcranial direct current stimulation, intermittent theta burst stimulation and high-frequency repetitive transcranial magnetic stimulation, high-frequency repetitive transcranial magnetic stimulation, and other non-invasive treatment modalities have played an important role in improving the prognosis of PSCI patients (Chen et al., 2023; Daoud et al., 2023; Ai et al., 2024). However, existing studies have not yet clarified their specific mechanism of action, and considering the important role of microglia in PSCI, it may be worthwhile to explore the association between the two in depth in the future. In addition to conventional drug treatment, Traditional Chinese medicine (TCM) treatment methods are currently receiving more and more attention and recognition from the world. Common TCM treatment methods include TCM and acupuncture. Recently, two meta-analyses found that TCM treatment plays a positive role in the treatment of PSCI (Cianciulli et al., 2015; Li et al., 2023). Previous studies have found that three TCMs, resveratrol, baicalein, and ginsenoside, can inhibit microglial inflammatory response by activating the JAK–STAT signaling pathway, inhibiting the NF-κB signaling pathway, and promoting M2-type anti-inflammation, respectively, which may be able to improve PSCI. Prognosis (Yang et al., 2019; Im, 2020; Liu et al., 2023). In addition to TCM treatment, acupuncture, as an ancient Chinese treatment method, also has certain clinical effects on PSCI (Ye et al., 2022). Domestic research in the field of TCM has found that acupuncture therapy between different acupoints can significantly reduce the expression of Bax and Fas, the key downstream target genes of the NF-κB pathway in microglia, and inhibit NF-κB-mediated neuronal cell activation. Apoptosis, thereby regulating the apoptotic pathway, inhibiting neuronal apoptosis, and improving cognitive impairment after stroke (Liu et al., 2021; Li et al., 2023). Although various drugs and TCM treatments currently bring new hope to the treatment of clinical patients. However, given that M1 and M2 microglia may appear in various stages of PSCI, TCM and acupuncture still lack reliable scientific basis. How to use drugs to regulate M1 microglia and explore acupuncture and its important mechanisms have become current difficulties. Considering that the active ingredients and mechanisms of action of most existing TCMs are still unclear. A large number of animal experiments and clinical studies are still needed to conduct more detailed mechanism studies to elucidate the potential therapeutic mechanism for PSCI.

## 6 Summary and future prospects

PSCI is a significant contributor to the overall burden of stroke disease and has emerged as a focal point in international stroke research, capturing widespread attention for clinical intervention. Ongoing research efforts are dedicated to cellular studies and the elucidation of mechanisms related to PSCI. Despite these efforts, substantial gaps remain in our comprehension of microglial biology, particularly concerning their mechanisms of action and the interplay among various signaling pathways. Future investigations should aim to further uncover the biological characteristics of microglia and delve into their underlying molecular mechanisms.

Moreover, existing studies underscore neuroinflammation as a pivotal mechanism in PSCI, with microglia playing a crucial role within this context. This review reveals that microglial activation can be triggered through multiple pathways, leading to the polarization of activated microglia into two distinct phenotypes: M1 and M2. These phenotypes exert divergent effects on PSCI, with M2 microglia serving a protective function, whereas M1 microglia contribute to detrimental outcomes. Identifying strategies to guide the polarization of microglia towards the M2 phenotype during PSCI progression represents a critical avenue for therapeutic intervention.

In concluding, this article accentuates the advancement of PSCI-related therapeutic approaches targeting microglia. However, the majority of these strategies are based on animal studies and retrospective clinical analyses with small sample sizes, lacking robust evidence from prospective, large-scale studies. Consequently, these treatment modalities remain a subject of debate. Moving forward, it is imperative to conduct prospective, randomized clinical trials across multiple centers to more accurately assess the impact of various treatment strategies on patients with PSCI.

## Author contributions

TZ: Writing – original draft, Writing – review & editing. JL: Writing – original draft, Writing – review & editing. WZ: Writing – original draft, Writing – review & editing. YY: Writing – review & editing. XY: Writing – review & editing. QH: Writing – original draft, Writing – review & editing. PL: Writing – original draft, Writing – review & editing. QJ: Writing – original draft, Writing – review & editing.

## References

[ref1] Ai Y. Liu Y. Yin M. Zhang L. Luo J. Zhang S.. (2024). Interactions between tDCS treatment and COMT Val158Met in poststroke cognitive impairment. Clin. Neurophysiol. 158, 43–55. doi: 10.1016/j.clinph.2023.12.011, PMID: 38176157

[ref2] Almarghalani D. A. Sha X. Mrak R. E. Shah Z. A. (2023). Spatiotemporal Cofilin signaling, microglial activation, Neuroinflammation, and cognitive impairment following hemorrhagic brain injury. Cells 12:1153. doi: 10.3390/cells12081153, PMID: 37190062 PMC10137307

[ref3] Alshammari A. Han Y. Jones T. W. Pillai B. Zhang D. Ergul A.. (2023). Stimulation of angiotensin II type 2 receptor modulates pro-inflammatory response in microglia and macrophages: therapeutic implications for the treatment of stroke. Life 13:1274. doi: 10.3390/life13061274, PMID: 37374057 PMC10302703

[ref4] Bahader G. A. Nash K. M. Almarghalani D. A. Alhadidi Q. McInerney M. F. Shah Z. A. (2021). Type-I diabetes aggravates post-hemorrhagic stroke cognitive impairment by augmenting oxidative stress and neuroinflammation in mice. Neurochem. Int. 149:105151. doi: 10.1016/j.neuint.2021.105151, PMID: 34348124 PMC8387457

[ref5] Barbay M. Diouf M. Roussel M. Godefroy O. GRECOGVASC study group (2018). Systematic review and Meta-analysis of prevalence in post-stroke neurocognitive disorders in hospital-based studies. Dement. Geriatr. Cogn. Disord. 46, 322–334. doi: 10.1159/000492920, PMID: 30504699

[ref6] Belevych N. Buchanan K. Chen Q. Bailey M. Quan N. (2010). Location-specific activation of the paraventricular nucleus of the hypothalamus by localized inflammation. Brain Behav. Immun. 24, 1137–1147. doi: 10.1016/j.bbi.2010.05.007, PMID: 20570615 PMC2939270

[ref7] Block M. L. Zecca L. Hong J. S. (2007). Microglia-mediated neurotoxicity: uncovering the molecular mechanisms. Nat. Rev. Neurosci. 8, 57–69. doi: 10.1038/nrn2038, PMID: 17180163

[ref8] Bond W. S. Rex T. S. (2014). Evidence that erythropoietin modulates Neuroinflammation through differential action on neurons, astrocytes, and microglia. Front. Immunol. 5:523. doi: 10.3389/fimmu.2014.00523, PMID: 25374571 PMC4205853

[ref9] Cavaliere F. Dinkel K. Reymann K. (2005). Microglia response and P2 receptor participation in oxygen/glucose deprivation-induced cortical damage. Neuroscience 136, 615–623. doi: 10.1016/j.neuroscience.2005.04.038, PMID: 16344139

[ref10] Chen S. Dong Z. Cheng M. Zhao Y. Wang M. Sai N.. (2017). Homocysteine exaggerates microglia activation and neuroinflammation through microglia localized STAT3 overactivation following ischemic stroke. J. Neuroinflammation 14:187. doi: 10.1186/s12974-017-0963-x, PMID: 28923114 PMC5604224

[ref11] Chen X. Xiu H. Hou Y. Chen X. Liu F. Tu S. (2023). High-frequency repetitive transcranial magnetic stimulation (HF-rTMS) on overall cognition in patients with post-stroke cognitive impairment: a systematic review and Meta-analysis. Am. J. Phys. Med. Rehabil. 103, 418–427. doi: 10.1097/PHM.0000000000002377 38113027

[ref12] Chu K. Yin B. Wang J. Peng G. Liang H. Xu Z.. (2012). Inhibition of P2X7 receptor ameliorates transient global cerebral ischemia/reperfusion injury via modulating inflammatory responses in the rat hippocampus. J. Neuroinflammation 9:69. doi: 10.1186/1742-2094-9-69, PMID: 22513224 PMC3418181

[ref13] Cianciulli A. Dragone T. Calvello R. Porro C. Trotta T. Lofrumento D. D.. (2015). IL-10 plays a pivotal role in anti-inflammatory effects of resveratrol in activated microglia cells. Int. Immunopharmacol. 24, 369–376. doi: 10.1016/j.intimp.2014.12.035, PMID: 25576658

[ref14] Colonna M. Butovsky O. (2017). Microglia function in the central nervous system during health and neurodegeneration. Annu. Rev. Immunol. 35, 441–468. doi: 10.1146/annurev-immunol-051116-052358, PMID: 28226226 PMC8167938

[ref15] Daoud A. Elsayed M. Alnajjar A. Z. Krayim A. AbdelMeseh M. Alsalloum T.. (2023). Efficacy of intermittent theta burst stimulation (iTBS) on post-stroke cognitive impairment (PSCI): a systematic review and meta-analysis. Neurol. Sci. 45, 2107–2118. doi: 10.1007/s10072-023-07267-w 38150130

[ref16] Davalos D. Grutzendler J. Yang G. Kim J. V. Zuo Y. Jung S.. (2005). ATP mediates rapid microglial response to local brain injury in vivo. Nat. Neurosci. 8, 752–758. doi: 10.1038/nn1472, PMID: 15895084

[ref17] Dong R. Huang R. Wang J. Liu H. Xu Z. (2021). Effects of microglial activation and polarization on brain injury after stroke. Front. Neurol. 12:620948. doi: 10.3389/fneur.2021.620948, PMID: 34276530 PMC8280287

[ref18] Dubbelaar M. L. Kracht L. Eggen B. J. L. Boddeke E. W. G. M. (2018). The kaleidoscope of microglial phenotypes. Front. Immunol. 9:1753. doi: 10.3389/fimmu.2018.01753, PMID: 30108586 PMC6079257

[ref19] Eglitis M. A. Mezey E. (1997). Hematopoietic cells differentiate into both microglia and macroglia in the brains of adult mice. Proc. Natl. Acad. Sci. USA 94, 4080–4085. doi: 10.1073/pnas.94.8.4080, PMID: 9108108 PMC20571

[ref20] Galic M. A. Riazi K. Pittman Q. J. (2012). Cytokines and brain excitability. Front. Neuroendocrinol. 33, 116–125. doi: 10.1016/j.yfrne.2011.12.002, PMID: 22214786 PMC3547977

[ref21] Gao C. Jiang J. Tan Y. Chen S. (2023). Microglia in neurodegenerative diseases: mechanism and potential therapeutic targets. Signal Transduct. Target. Ther. 8:359. doi: 10.1038/s41392-023-01588-0 37735487 PMC10514343

[ref22] Ge Y. Yang J. Chen J. Dai M. Dou X. Yao S.. (2024). Absence in CX3CR1 receptor signaling promotes post-ischemic stroke cognitive function recovery through suppressed microglial pyroptosis in mice. CNS Neurosci. Ther. 30:e14551. doi: 10.1111/cns.14551, PMID: 38421089 PMC10850801

[ref23] Ginhoux F. Greter M. Leboeuf M. Nandi S. See P. Gokhan S.. (2010). Fate mapping analysis reveals that adult microglia derive from primitive macrophages. Science 330, 841–845. doi: 10.1126/science.1194637 20966214 PMC3719181

[ref24] Gülke E. Gelderblom M. Magnus T. (2018). Danger signals in stroke and their role on microglia activation after ischemia. Ther. Adv. Neurol. Disord. 11:1756286418774254. doi: 10.1177/1756286418774254, PMID: 29854002 PMC5968660

[ref25] Hait N. C. Wise L. E. Allegood J. C. O'Brien M. Avni D. Reeves T. M.. (2014). Active, phosphorylated fingolimod inhibits histone deacetylases and facilitates fear extinction memory. Nat. Neurosci. 17, 971–980. doi: 10.1038/nn.3728 24859201 PMC4256678

[ref26] Hirayama Y. Anzai N. Koizumi S. (2021). Mechanisms underlying sensitization of P2X7 receptors in astrocytes for induction of ischemic tolerance. Glia 69, 2100–2110. doi: 10.1002/glia.23998, PMID: 34076906

[ref27] Hoogland I. C. Houbolt C. van Westerloo D. J. van Gool W. A. van de Beek D. (2015). Systemic inflammation and microglial activation: systematic review of animal experiments. J. Neuroinflammation 12:114. doi: 10.1186/s12974-015-0332-6, PMID: 26048578 PMC4470063

[ref28] Hu X. Li P. Guo Y. Wang H. Leak R. K. Chen S.. (2012). Microglia/macrophage polarization dynamics reveal novel mechanism of injury expansion after focal cerebral ischemia. Stroke 43, 3063–3070. doi: 10.1161/STROKEAHA.112.659656, PMID: 22933588

[ref29] Huang Y. Wang Q. Zou P. He G. Zeng Y. Yang J. (2023). Prevalence and factors influencing cognitive impairment among the older adult stroke survivors: a cross-sectional study. Front. Public Health 11:1254126. doi: 10.3389/fpubh.2023.1254126, PMID: 37790718 PMC10542404

[ref30] Illes P. Ulrich H. Chen J. F. Tang Y. (2023). Purinergic receptors in cognitive disturbances. Neurobiol. Dis. 185:106229. doi: 10.1016/j.nbd.2023.106229, PMID: 37453562

[ref31] Im D. S. (2020). Pro-resolving effect of Ginsenosides as an anti-inflammatory mechanism of *Panax ginseng*. Biomol. Ther. 10:444. doi: 10.3390/biom10030444, PMID: 32183094 PMC7175368

[ref32] Jackson L. Dumanli S. Johnson M. H. Fagan S. C. Ergul A. (2020). Microglia knockdown reduces inflammation and preserves cognition in diabetic animals after experimental stroke. J. Neuroinflammation 17:137. doi: 10.1186/s12974-020-01815-3, PMID: 32345303 PMC7189436

[ref33] Jiang Z. Wei J. Liang J. Huang W. Ouyang F. Chen C.. (2024). Dl-3-n-butylphthalide alleviates secondary brain damage and improves working memory after stroke in Cynomolgus monkeys. Stroke 55, 725–734. doi: 10.1161/STROKEAHA.123.045037, PMID: 38406851

[ref34] Lecca D. Jung Y. J. Scerba M. T. Hwang I. Kim Y. K. Kim S.. (2022). Role of chronic neuroinflammation in neuroplasticity and cognitive function: a hypothesis. Alzheimers Dement. 18, 2327–2340. doi: 10.1002/alz.12610, PMID: 35234334 PMC9437140

[ref35] Lehnardt S. (2010). Innate immunity and neuroinflammation in the CNS: the role of microglia in toll-like receptor-mediated neuronal injury. Glia 58, 253–263. doi: 10.1002/glia.20928, PMID: 19705460

[ref36] Li Y. Cui R. Liu S. Qin Z. Sun W. Cheng Y.. (2023). The efficacy and safety of post-stroke cognitive impairment therapies: an umbrella review. Front. Pharmacol. 14:1207075. doi: 10.3389/fphar.2023.1207075, PMID: 37693907 PMC10483224

[ref37] Li Y. Liao J. Xiong L. Xiao Z. Ye F. Wang Y.. (2024). Stepwise targeted strategies for improving neurological function by inhibiting oxidative stress levels and inflammation following ischemic stroke. J. Control. Release 368, 607–622. doi: 10.1016/j.jconrel.2024.02.039, PMID: 38423472

[ref38] Li T. Pang S. Yu Y. Wu X. Guo J. Zhang S. (2013). Proliferation of parenchymal microglia is the main source of microgliosis after ischaemic stroke. Brain 136, 3578–3588. doi: 10.1093/brain/awt287, PMID: 24154617

[ref39] Li Y. F. Ren X. Zhang L. Wang Y. H. Chen T. (2022). Microglial polarization in TBI: signaling pathways and influencing pharmaceuticals. Front. Aging Neurosci. 14:901117. doi: 10.3389/fnagi.2022.901117, PMID: 35978950 PMC9376354

[ref40] Li N. Wang H. Liu H. Zhu L. Lyu Z. Qiu J.. (2023). The effects and mechanisms of acupuncture for post-stroke cognitive impairment: progress and prospects. Front. Neurosci. 17:1211044. doi: 10.3389/fnins.2023.1211044, PMID: 37397457 PMC10309044

[ref41] Li F. Xu D. Hou K. Gou X. Li Y. (2020). The role of P2Y12 receptor inhibition in ischemic stroke on microglia, platelets and vascular smooth muscle cells. J. Thromb. Thrombolysis 50, 874–885. doi: 10.1007/s11239-020-02098-4, PMID: 32248335

[ref42] Liu J. Ma W. Zang C. H. Wang G. D. Zhang S. J. Wu H. J.. (2021). Salidroside inhibits NLRP3 inflammasome activation and apoptosis in microglia induced by cerebral ischemia/reperfusion injury by inhibiting the TLR4/NF-κB signaling pathway. Ann. Transl. Med. 9:1694. doi: 10.21037/atm-21-5752, PMID: 34988203 PMC8667139

[ref43] Liu X. Nemeth D. P. Tarr A. J. Belevych N. Syed Z. W. Wang Y.. (2016). Euflammation attenuates peripheral inflammation-induced neuroinflammation and mitigates immune-to-brain signaling. Brain Behav. Immun. 54, 140–148. doi: 10.1016/j.bbi.2016.01.018, PMID: 26812118 PMC4828265

[ref44] Liu Z. J. Ran Y. Y. Qie S. Y. Gong W. J. Gao F. H. Ding Z. T.. (2019). Melatonin protects against ischemic stroke by modulating microglia/macrophage polarization toward anti-inflammatory phenotype through STAT3 pathway. CNS Neurosci. Ther. 25, 1353–1362. doi: 10.1111/cns.13261, PMID: 31793209 PMC6887673

[ref45] Liu Y. Zhao L. Chen F. Li X. Han J. Sun X.. (2023). Comparative efficacy and safety of multiple acupuncture therapies for post stroke cognitive impairment: a network meta-analysis of randomized controlled trials. Front. Neurol. 14:1218095. doi: 10.3389/fneur.2023.1218095, PMID: 37638181 PMC10447897

[ref46] Lou N. Takano T. Pei Y. Xavier A. L. Goldman S. A. Nedergaard M. (2016). Purinergic receptor P2RY12-dependent microglial closure of the injured blood-brain barrier. Proc. Natl. Acad. Sci. USA 113, 1074–1079. doi: 10.1073/pnas.1520398113, PMID: 26755608 PMC4743790

[ref47] Lu Y. Zhou M. Li Y. Li Y. Hua Y. Fan Y. (2021). Minocycline promotes functional recovery in ischemic stroke by modulating microglia polarization through STAT1/STAT6 pathways. Biochem. Pharmacol. 186:114464. doi: 10.1016/j.bcp.2021.114464, PMID: 33577892

[ref48] Masuda T. Croom D. Hida H. Kirov S. A. (2011). Capillary blood flow around microglial somata determines dynamics of microglial processes in ischemic conditions. Glia 59, 1744–1753. doi: 10.1002/glia.21220, PMID: 21800362 PMC3174346

[ref49] Matsunaga N. Tsuchimori N. Matsumoto T. Ii M. (2011). TAK-242 (resatorvid), a small-molecule inhibitor of toll-like receptor (TLR) 4 signaling, binds selectively to TLR4 and interferes with interactions between TLR4 and its adaptor molecules. Mol. Pharmacol. 79, 34–41. doi: 10.1124/mol.110.068064, PMID: 20881006

[ref50] Meng H. L. Li X. X. Chen Y. T. Yu L. J. Zhang H. Lao J. M.. (2016). Neuronal soluble Fas ligand drives M1-microglia polarization after cerebral ischemia. CNS Neurosci. Ther. 22, 771–781. doi: 10.1111/cns.12575, PMID: 27283206 PMC6492913

[ref51] Miron J. Picard C. Frappier J. Dea D. Théroux L. Poirier J. (2018). TLR4 gene expression and pro-inflammatory cytokines in Alzheimer's disease and in response to hippocampal Deafferentation in rodents. J. Alzheimer's Dis. 63, 1547–1556. doi: 10.3233/JAD-171160, PMID: 29782315

[ref52] Moretti R. Leger P. L. Besson V. C. Csaba Z. Pansiot J. Di Criscio L.. (2016). Sildenafil, a cyclic GMP phosphodiesterase inhibitor, induces microglial modulation after focal ischemia in the neonatal mouse brain. J. Neuroinflammation 13:95. doi: 10.1186/s12974-016-0560-4, PMID: 27126393 PMC4850658

[ref53] Morrison H. W. Filosa J. A. (2013). A quantitative spatiotemporal analysis of microglia morphology during ischemic stroke and reperfusion. J. Neuroinflammation 10:4. doi: 10.1186/1742-2094-10-4, PMID: 23311642 PMC3570327

[ref54] Morrone F. B. Vargas P. Rockenbach L. Scheffel T. B. (2021). P2Y12 purinergic receptor and brain tumors: implications on glioma microenvironment. Molecules 26:6146. doi: 10.3390/molecules26206146, PMID: 34684726 PMC8540665

[ref55] Mut-Arbona P. Sperlágh B. (2023). P2 receptor-mediated signaling in the physiological and pathological brain: from development to aging and disease. Neuropharmacology 233:109541. doi: 10.1016/j.neuropharm.2023.109541, PMID: 37062423

[ref56] Nair D. Dayyat E. A. Zhang S. X. Wang Y. Gozal D. (2011). Intermittent hypoxia-induced cognitive deficits are mediated by NADPH oxidase activity in a murine model of sleep apnea. PLoS One 6:e19847. doi: 10.1371/journal.pone.0019847, PMID: 21625437 PMC3100309

[ref57] Nayak D. Roth T. L. McGavern D. B. (2014). Microglia development and function. Annu. Rev. Immunol. 32, 367–402. doi: 10.1146/annurev-immunol-032713-120240, PMID: 24471431 PMC5001846

[ref58] Niu F. N. Zhang X. Hu X. M. Chen J. Chang L. L. Li J. W.. (2012). Targeted mutation of Fas ligand gene attenuates brain inflammation in experimental stroke. Brain Behav. Immun. 26, 61–71. doi: 10.1016/j.bbi.2011.07.235, PMID: 21802508

[ref59] Paul M. Paul J. W. Hinwood M. Hood R. J. Martin K. Abdolhoseini M.. (2023). Clopidogrel administration impairs post-stroke learning and memory recovery in mice. Int. J. Mol. Sci. 24:11706. doi: 10.3390/ijms241411706, PMID: 37511466 PMC10380815

[ref60] Pawate S. Shen Q. Fan F. Bhat N. R. (2004). Redox regulation of glial inflammatory response to lipopolysaccharide and interferongamma. J. Neurosci. Res. 77, 540–551. doi: 10.1002/jnr.20180, PMID: 15264224

[ref61] Perego C. Fumagalli S. De Simoni M. G. (2011). Temporal pattern of expression and colocalization of microglia/macrophage phenotype markers following brain ischemic injury in mice. J. Neuroinflammation 8:174. doi: 10.1186/1742-2094-8-174, PMID: 22152337 PMC3251548

[ref62] Prinz M. Jung S. Priller J. (2019). Microglia biology: one century of evolving concepts. Cell 179, 292–311. doi: 10.1016/j.cell.2019.08.053, PMID: 31585077

[ref63] Qin S. Zhang Z. Zhao Y. Liu J. Qiu J. Gong Y.. (2022). The impact of acupuncture on neuroplasticity after ischemic stroke: a literature review and perspectives. Front. Cell. Neurosci. 16:817732. doi: 10.3389/fncel.2022.817732, PMID: 36439200 PMC9685811

[ref64] Quan N. (2014). In-depth conversation: spectrum and kinetics of neuroimmune afferent pathways. Brain Behav. Immun. 40, 1–8. doi: 10.1016/j.bbi.2014.02.006, PMID: 24566385 PMC6088807

[ref65] Rajan W. D. Wojtas B. Gielniewski B. Gieryng A. Zawadzka M. Kaminska B. (2019). Dissecting functional phenotypes of microglia and macrophages in the rat brain after transient cerebral ischemia. Glia 67, 232–245. doi: 10.1002/glia.23536, PMID: 30485549

[ref66] Ricci A. Liesz A. (2023). A tale of two cells: regulatory T cell-microglia cross-talk in the ischemic brain. Sci. Transl. Med. 15:eadj0052. doi: 10.1126/scitranslmed.adj0052 37939163

[ref67] Ronaldson P. T. Davis T. P. (2020). Regulation of blood-brain barrier integrity by microglia in health and disease: a therapeutic opportunity. J. Cereb. Blood Flow Metab. 40, S6–S24. doi: 10.1177/0271678X20951995, PMID: 32928017 PMC7687032

[ref68] Roy A. Jana A. Yatish K. Freidt M. B. Fung Y. K. Martinson J. A.. (2008). Reactive oxygen species up-regulate CD11b in microglia via nitric oxide: implications for neurodegenerative diseases. Free Radic. Biol. Med. 45, 686–699. doi: 10.1016/j.freeradbiomed.2008.05.026, PMID: 18590811 PMC2701551

[ref69] Satriotomo I. Bowen K. K. Vemuganti R. (2006). JAK2 and STAT3 activation contributes to neuronal damage following transient focal cerebral ischemia. J. Neurochem. 98, 1353–1368. doi: 10.1111/j.1471-4159.2006.04051.x, PMID: 16923154

[ref70] Sayed M. A. Eldahshan W. Abdelbary M. Pillai B. Althomali W. Johnson M. H.. (2020). Stroke promotes the development of brain atrophy and delayed cell death in hypertensive rats. Sci. Rep. 10:20233. doi: 10.1038/s41598-020-75450-6, PMID: 33214598 PMC7678843

[ref71] Skrobot O. A. O'Brien J. Black S. Chen C. DeCarli C. Erkinjuntti T.. (2017). The vascular impairment of cognition classification consensus study. Alzheimers Dement. 13, 624–633. doi: 10.1016/j.jalz.2016.10.007, PMID: 27960092

[ref72] Socodato R. Portugal C. C. Domith I. Oliveira N. A. Coreixas V. S. Loiola E. C.. (2015). C-Src function is necessary and sufficient for triggering microglial cell activation. Glia 63, 497–511. doi: 10.1002/glia.22767 25421817

[ref73] Tack R. W. P. Amboni C. van Nuijs D. Pekna M. Vergouwen M. D. I. Rinkel G. J. E.. (2023). Inflammation, anti-inflammatory interventions, and post-stroke cognitive impairment: a systematic review and Meta-analysis of human and animal studies. Transl. Stroke Res. doi: 10.1007/s12975-023-01218-5, PMID: 38012509 PMC11976800

[ref74] Teng Z. Dong Y. Zhang D. An J. Lv P. (2017). Cerebral small vessel disease and post-stroke cognitive impairment. Int. J. Neurosci. 127, 824–830. doi: 10.1080/00207454.2016.1261291 27838946

[ref75] Vidyanti A. N. Hsieh J. Y. Lin K. J. Fang Y. C. Setyopranoto I. Hu C. J. (2020). Role of HMGB1 in an animal model of vascular cognitive impairment induced by chronic cerebral Hypoperfusion. Int. J. Mol. Sci. 21:2176. doi: 10.3390/ijms21062176, PMID: 32245271 PMC7139598

[ref76] Volonté C. Amadio S. D'Ambrosi N. Colpi M. Burnstock G. (2006). P2 receptor web: complexity and fine-tuning. Pharmacol. Ther. 112, 264–280. doi: 10.1016/j.pharmthera.2005.04.012, PMID: 16780954

[ref77] Wang K. Dong Q. (2021). Experts consensus on post-stroke cognitive impairment management 2021. Chin. J. Stroke 16, 376–389. doi: 10.3969/j.issn.1673-5765.2021.04.011

[ref78] Wang Z. Q. Li K. Huang J. Huo T. T. Lv P. Y. (2021). Corrigendum: MicroRNA let-7i is a promising serum biomarker for post-stroke cognitive impairment and alleviated OGD-induced cell damage in vitro by regulating Bcl-2. Front. Neurosci. 15:648121. doi: 10.3389/fnins.2021.648121, PMID: 33597845 PMC7883378

[ref79] Wang H. Zhang M. Li J. Liang J. Yang M. Xia G.. (2022). Gut microbiota is causally associated with poststroke cognitive impairment through lipopolysaccharide and butyrate. J. Neuroinflammation 19:76. doi: 10.1186/s12974-022-02435-9, PMID: 35379265 PMC8981610

[ref80] Wang R. Zhang S. Yang Z. Zheng Y. Yan F. Tao Z.. (2021). Mutant erythropoietin enhances white matter repair via the JAK2/STAT3 and C/EBPβ pathway in middle-aged mice following cerebral ischemia and reperfusion. Exp. Neurol. 337:113553. doi: 10.1016/j.expneurol.2020.113553 33309747

[ref81] Wang Y. Zhu Y. Wang J. Dong L. Liu S. Li S.. (2023). Purinergic signaling: a gatekeeper of blood-brain barrier permeation. Front. Pharmacol. 14:1112758. doi: 10.3389/fphar.2023.1112758, PMID: 36825149 PMC9941648

[ref82] Xiong X. Y. Liu L. Yang Q. W. (2016). Functions and mechanisms of microglia/macrophages in neuroinflammation and neurogenesis after stroke. Prog. Neurobiol. 142, 23–44. doi: 10.1016/j.pneurobio.2016.05.001, PMID: 27166859

[ref83] Xu L. He D. Bai Y. (2016). Microglia-mediated inflammation and neurodegenerative disease. Mol. Neurobiol. 53, 6709–6715. doi: 10.1007/s12035-015-9593-4 26659872

[ref84] Yang C. Hawkins K. E. Doré S. Candelario-Jalil E. (2019). Neuroinflammatory mechanisms of blood-brain barrier damage in ischemic stroke. Am. J. Physiol. Cell Physiol. 316, C135–C153. doi: 10.1152/ajpcell.00136.2018 30379577 PMC6397344

[ref85] Yang S. Wang H. Yang Y. Wang R. Wang Y. Wu C.. (2019). Baicalein administered in the subacute phase ameliorates ischemia-reperfusion-induced brain injury by reducing neuroinflammation and neuronal damage. Biomed. Pharmacother. 117:109102. doi: 10.1016/j.biopha.2019.109102, PMID: 31228802

[ref86] Yang H. C. Zhang M. Wu R. Zheng H. Q. Zhang L. Y. Luo J.. (2020). C-C chemokine receptor type 2-overexpressing exosomes alleviated experimental post-stroke cognitive impairment by enhancing microglia/macrophage M2 polarization. World J. Stem Cells 12, 152–167. doi: 10.4252/wjsc.v12.i2.152, PMID: 32184939 PMC7062036

[ref87] Ye M. Zheng Y. Xiong Z. Ye B. Zheng G. (2022). Baduanjin exercise ameliorates motor function in patients with post-stroke cognitive impairment: a randomized controlled trial. Complement. Ther. Clin. Pract. 46:101506. doi: 10.1016/j.ctcp.2021.101506, PMID: 34742096

[ref88] Yu J. Zhao Y. Gong X. K. Liang Z. Zhao Y. N. Li X.. (2023). P25/CDK5-mediated tau hyperphosphorylation in both ipsilateral and contralateral Cerebra contributes to cognitive deficits in post-stroke mice. Curr. Med. Sci. 43, 1084–1095. doi: 10.1007/s11596-023-2792-8, PMID: 37924385

[ref89] Zhang S. (2019). Microglial activation after ischaemic stroke. Stroke Vasc. Neurol. 4, 71–74. doi: 10.1136/svn-2018-000196 31338213 PMC6613941

[ref90] Zhang H. Wang L. Yang Y. Cai C. Wang X. Deng L.. (2023). DL-3-n-butylphthalide (NBP) alleviates poststroke cognitive impairment (PSCI) by suppressing neuroinflammation and oxidative stress. Front. Pharmacol. 13:987293. doi: 10.3389/fphar.2022.987293, PMID: 36712684 PMC9878832

[ref91] Zhu G. Wang X. Chen L. Lenahan C. Fu Z. Fang Y.. (2022). Crosstalk between the oxidative stress and glia cells after stroke: from mechanism to therapies. Front. Immunol. 13:852416. doi: 10.3389/fimmu.2022.852416, PMID: 35281064 PMC8913707

[ref92] Zou C. G. Zhao Y. S. Gao S. Y. Li S. D. Cao X. Z. Zhang M.. (2010). Homocysteine promotes proliferation and activation of microglia. Neurobiol. Aging 31, 2069–2079. doi: 10.1016/j.neurobiolaging.2008.11.007, PMID: 19131143

